# Translational regulation contributes to the secretory response of chondrocytic cells following exposure to interleukin-1β

**DOI:** 10.1074/jbc.RA118.006865

**Published:** 2019-07-12

**Authors:** Benjamin T. McDermott, Mandy J. Peffers, Brian McDonagh, Simon R. Tew

**Affiliations:** ‡ Department of Musculoskeletal Biology, Institute of Ageing and Chronic Disease, University of Liverpool, William Henry Duncan Building, 6 West Derby Street, Liverpool L7 8TX, United Kingdom; § Department of Physiology, School of Medicine, National University of Ireland (NUI), Galway H91 TK33, Ireland

**Keywords:** chondrocyte, inflammation, interleukin 1 (IL-1), osteoarthritis, post-transcriptional regulation, proteomics, superoxide dismutase (SOD)

## Abstract

Osteoarthritis is a chronic disease characterized by the loss of articular cartilage in synovial joints through a process of extracellular matrix destruction that is strongly associated with inflammatory stimuli. Chondrocytes undergo changes to their protein translational capacity during osteoarthritis, but a study of how disease-relevant signals affect chondrocyte protein translation at the transcriptomic level has not previously been performed. In this study, we describe how the inflammatory cytokine interleukin 1-β (IL-1β) rapidly affects protein translation in the chondrocytic cell line SW1353. Using ribosome profiling we demonstrate that IL-1β induced altered translation of inflammatory-associated transcripts such as NFKB1, TNFAIP2, MMP13, CCL2, and CCL7, as well as a number of ribosome-associated transcripts, through differential translation and the use of multiple open reading frames. Proteomic analysis of the cellular layer and the conditioned media of these cells identified changes in a number of the proteins that were differentially translated. Translationally regulated secreted proteins included a number of chemokines and cytokines, underlining the rapid, translationally mediated inflammatory cascade that is initiated by IL-1β. Although fewer cellular proteins were found to be regulated in both ribosome profiling and proteomic data sets, we did find increased levels of SOD2, indicative of redox changes within SW1353 cells being modulated at the translational level. In conclusion, we have produced combined ribosome profiling and proteomic data sets that provide a valuable resource in understanding the processes that occur during cytokine stimulation of chondrocytic cells.

## Introduction

Osteoarthritis (OA)^2^ is a chronic disease characterized by the loss of articular cartilage in synovial joints through a process of extracellular matrix destruction, driven by extracellular proteases (1, 2). The disease is multifactorial, and risk factors include age, genetic predisposition, prior trauma, and obesity (3–5). Once considered to be driven primarily by mechanical processes, it is becoming apparent that there is a significant role for inflammation in the pathophysiology of the disease (6, 7). The expression of inflammatory cytokines such as interleukin 1-β (IL-1β) and tumor necrosis factor-α (TNFα) caused by excessive mechanical loading and trauma can contribute to the onset of inflammation and alterations to the articular cartilage due to the promoted actions of catabolic proteases such as members of the matrix metalloproteinase (MMP) family (8–11). Cytokines are also able to induce catabolic gene expression in chondrocyte cell lines *in vitro*, mimicking many of the processes that occur in chondrocytes during OA development, making them valuable model systems for determining disease mechanisms (12, 13). The stable phenotype of a cell line for these *in vitro* studies makes it particularly useful for techniques where large numbers of cells are required, which would be limited by the use of primary cells. However, when interpreting data from these studies it is important that we focus our interpretation on processes where the cell line best reflects primary cell function.

Changes in the steady-state levels of a transcript and its translation rate are not always well-correlated (14–17). For some time, studies focusing on specific genes have provided evidence for this, and more recently, advances in ribosome footprinting approaches have confirmed this disassociation (18–21). Marker gene expression is often used as a proxy for functional phenotype in disease studies and has led to a significant increase in our understanding of how regulators of disease control the transcriptional landscapes. Although the steady-state mRNA levels measured in transcriptomic studies provide important information on the modulation of cellular phenotype under different conditions, they fail to fully inform us about the dynamic post-transcriptional control of mRNA turnover and translation. Such processes represent a vital tier of phenotypic regulation and contribute substantially to the overall functional landscape of the cell.

A number of informative studies have described transcriptomic profiling of cells from healthy and diseased cartilage (22–25) as well as in cell-based models of chondrocyte inflammatory responses (26, 27). More recently, our group has demonstrated transcriptome-wide differences in the rates of mRNA decay in chondrocytes from normal and osteoarthritic tissues (24) and have also shown how post-transcriptional regulation via RNA-binding proteins affects both anabolic and catabolic gene expression in human chondrocytes (28). These studies coincide with the emerging role for microRNAs in chondrocyte biology, with links among post-transcriptional control of key cartilage-degrading proteinases in animal models, human disease, and aging (29–32). Although it is still unclear to what extent discrete control of mRNA translation is involved in the processes that drive OA, there is emerging evidence that suggests that global translation is significantly altered by the disease (33).

Ribosomal profiling is an exciting methodology that utilizes RNA-Seq technology to analyze the ribosomal footprints of cellular mRNAs, providing transcriptome-wide resolution of active translation within a cell at a given time. This technique has already led to a number of fresh insights into the mechanisms underlying translation across species as well as identifying previously unrecognized protein isoforms (34–37). A current requirement for this technique is the relatively large starting quantity of RNA required per sample as only 5–20% of input RNA is recovered after the ribosomal RNA (rRNA) removal step, making analysis of many primary tissue sources extremely challenging. Therefore, in this study, we have chosen to use ribosomal profiling and proteomic approaches to examine how translation is altered in an *in vitro* model of chondrocyte response using the SW1353 chondrosarcoma line stimulated with the inflammatory cytokine IL-1β. By examining total mRNA levels and ribosome-protected mRNA levels and by performing parallel proteomic analysis, we provide new understanding of how IL-1β regulates genes as well as identify specific groups of mRNAs where translational and transcriptional level do not necessarily correlate.

## Results

### Ribosome-protected mRNA suitable for RNA-Seq analysis can be isolated from SW1353 cells

**Figure 1.**
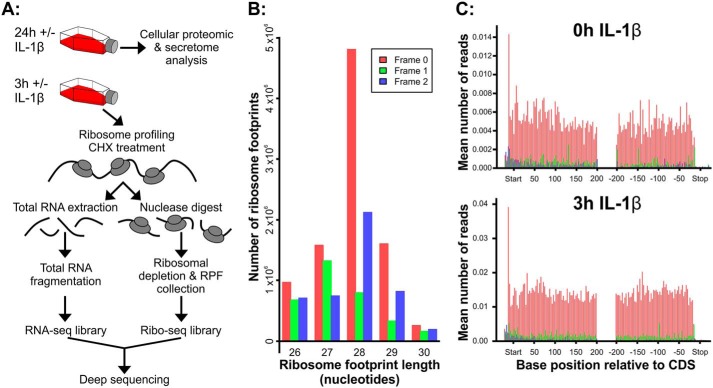
**Ribosome profiling workflow and sequencing data.** *A*, SW1353 workflow. Cells were treated for 3 or 24 h with or without IL-1β. Medium was harvested for secretome proteomic analysis, and cells were harvested for intracellular proteomic analysis after 24-h IL-1β exposure. Ribosome profiling was performed on cells exposed to IL-1β for 0 and 3 h. *B*, to obtain triplet periodicity of ribosome-protected fragments, sequencing results (FASTQ files) from amplified libraries were first uploaded to RiboGalaxy. The 5′-AGATCGGAAGAGCACACGTCT-3′ adaptor sequence and rRNA sequences were removed using Cutadapt and Bowtie. Sequences were then aligned to the human transcriptome (GRCh38/hg38) using Bowtie prior to riboSeqR analysis. From the riboSeqR analysis, triplet periodicity showed that most sequencing reads were 28 nucleotides in length and in frame 1 (*red*). *C*, RiboGalaxy was next used to perform a metagene analysis of the ribosome density and showed similar ribosomal coverage over all transcripts that are 28 nucleotides in length. This representative metagene analysis reflects triplet periodicity results in that the majority of 28-mer transcripts are in frame 1 (*red*). *CHX*, cycloheximide.

To determine how IL-1β affected translation rates, we performed ribosome profiling analysis on SW1353 cells exposed to the cytokine for 3 h. Total and ribosome-protected RNA from IL-1β–treated SW1353 cell cultures was isolated and processed for RNA-Seq analysis (Fig. 1*A*; extended workflow shown in Fig. S1). Bioanalyzer analysis of RNA-Seq libraries showed amplicons with the expected size distributions for each sample (Table S3). Following sequencing, reads were processed and then aligned to the human genome before differential translation analysis was performed using the riboSeqR code via the web-based RiboGalaxy interface. A detailed breakdown of all RiboGalaxy parameters is shown in Table S4. Analysis of triplet periodicity in the ribosomal profiled samples demonstrated that the reads were mostly 28-mers with enrichment for those in frame 1, indicating that those reads were in-frame with the coded protein. (Fig. 1*B*). Metagene analysis using the plotCDS function confirmed that the 28-mer reads were predominantly frame 1 and demonstrated that they were evenly aligned at both the start and end of protein coding regions with a peak in reads at the translation start site and a small decrease in reads toward the stop codons (Fig. 1*C*).

### Ribosome profiling demonstrates that IL-1β–induced inflammatory secretory proteins in SW1353 cells are differentially translated

**Figure 2.**
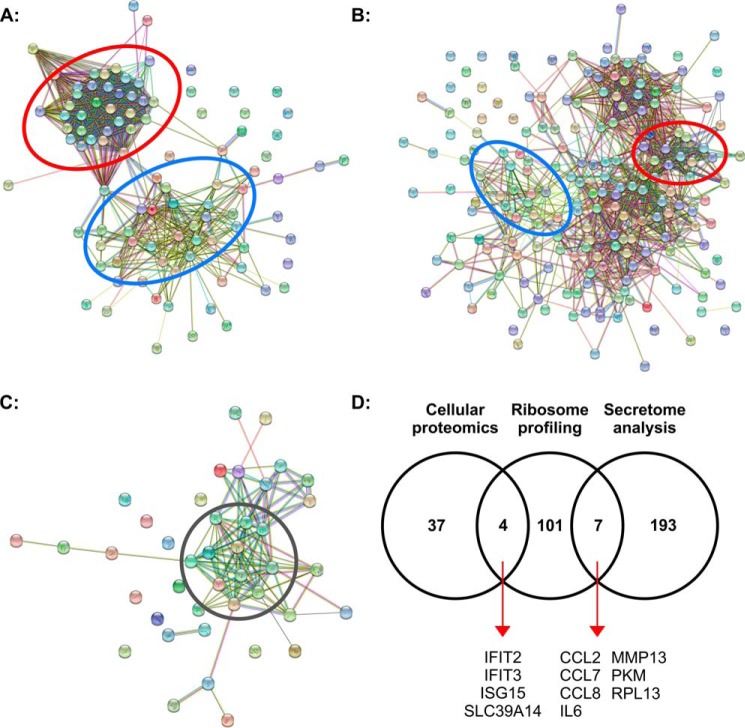
**SW1353 transcript/proteomic analyses.** *A*, STRING analysis of the 112 transcripts of statistically significant differentially translated RPFs from the 3-h ±IL-1β treatment. *B* and *C*, STRING analysis of the 200 statistically significant differentially secreted proteins from the 24-h ±IL-1β treatment (*B*) and the 41 statistically significant differentially expressed proteins identified in the cell-layer proteomic analysis (*C*). *D*, Venn diagram showing the relationship of the top 200 transcripts/proteins identified through cellular proteomic analysis, ribosome profiling, and secretome analysis. *Red circles* highlight clustering of ribosome-associated transcripts/proteins, and *blue circles* highlight clustering of inflammatory-associated transcripts/proteins. The *gray circle* highlights interferon-associated proteins. A full list of transcripts/proteins and the STRING networks with labels can be found in the supporting information tables.

Differential translation analysis of ribosome-protected fragments (RPFs) was performed using the baySeq (38) function via RiboGalaxy and revealed that the majority of the statistically significant (*q* < 0.001) top 200 transcripts differentially translated following IL-1β stimulation were increased compared with untreated (Table S5). Once duplicate transcripts were excluded, there were 112 transcripts for individual proteins. Only four transcripts translated at a lower rate: ZC3H12A, two serine/threonine kinase PIM1 transcripts, and one of two transcript variants for TNFAIP2. The majority of the differentially translated transcripts were identified as linear mRNA transcripts (93% of transcripts) with the remaining identified as noncoding RNAs or pseudogenes (7% of transcripts; Table S5). STRING analysis of the top 200 differentially translated transcripts identified via ribosome profiling demonstrates that a large proportion coded for either inflammatory-associated proteins (Fig. 2*A*, *blue circle*; 37 transcripts; 18.5%) or ribosome-associated proteins (Fig. 2*A*, *red circle*; 63 transcripts; 31.5%). Interestingly, STRING-derived protein networks from the top 200 statistically significant (*q* < 0.05) proteins identified in the secretome data showed similar groupings (Fig. 2*B*). STRING analysis of the 42 proteins significantly differentially regulated (*q* < 0.05) in the cellular proteomic analysis identified a cluster of interferon-associated proteins regulated 24 h after IL-1β stimulation (Fig. 2*C*, *gray circle*). Commonality between these significantly regulated transcripts/proteins identified by ribosome profiling, cellular proteomic analysis, and secretome analysis is illustrated in Fig. 2*D*. A number of proteins that were observed to be differentially translated after 3-h exposure to IL-1β also exhibited alterations in their absolute level after 24-h exposure with examples observed in both the cellular proteome (IFIT2/3 ISG15 and SLC39A14) and in the secretome (CCL2, CCL7, CCL8, IL-6, MMP13, PKM, and RPL13). Taken together, our data suggest that a proportion of the inflammatory response of SW1353 cells to IL-1β treatment occurs as a result of the rapid increase in the translation rates of chemokines, cytokines, and associated secretory proteins.

**Figure 3.**
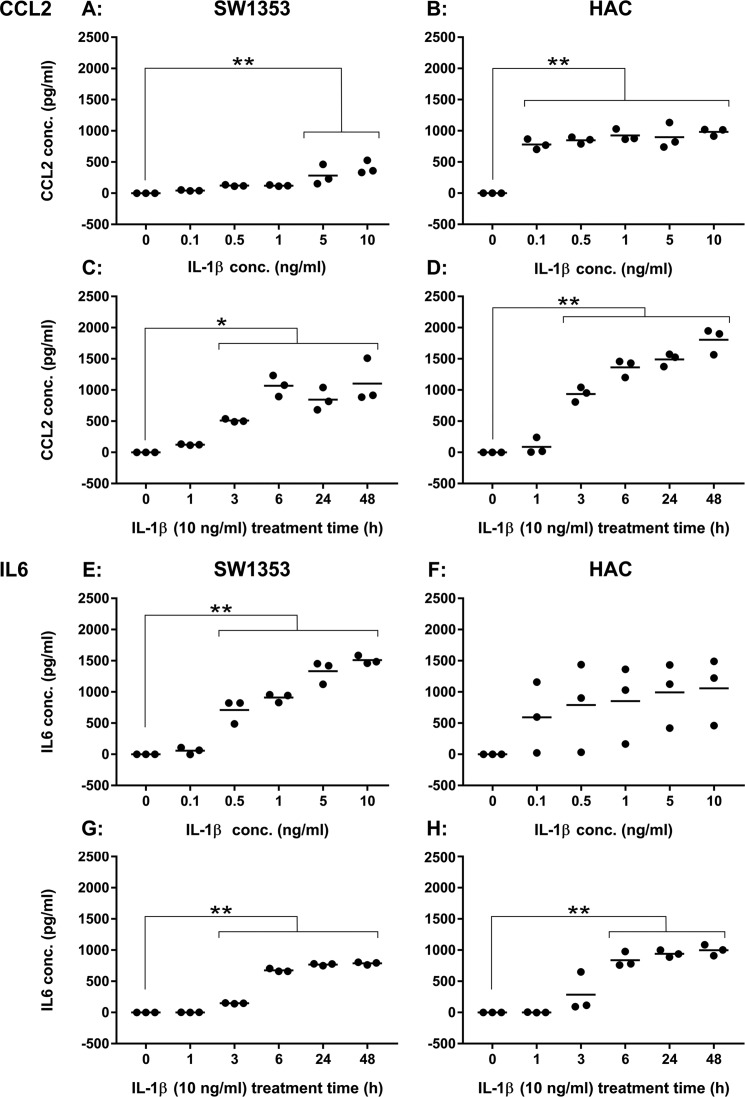
**Cytokine secretion levels following exposure to IL-1β.** *A*, CCL2 levels in SW1353 medium following 24-h exposure to IL-1β at concentrations ranging from 0 to 10 ng/ml. *B*, CCL2 levels in HAC (M70, M76, and M86) medium following 24-h exposure to IL-1β at concentrations ranging from 0 to 10 ng/ml. *C*, CCL2 levels in SW1353 medium following 10 ng/ml IL-1β treatment for 0–48 h. *D*, CCL2 levels in HAC (M69, F80, and F86) medium following 10 ng/ml IL-1β treatment for 0–48 h. *E*, IL-6 levels in SW1353 medium following 24-h exposure to IL-1β at concentrations ranging from 0 to 10 ng/ml. *F*, IL-6 levels in HAC (M70, M76, and M86) medium following 24-h exposure to IL-1β at concentrations ranging from 0 to 10 ng/ml. *G*, IL-6 levels in SW1353 medium following 10 ng/ml IL-1β treatment for 0–48 h. *H*, IL-6 levels in HAC (M69, F80, and F86) medium following 10 ng/ml IL-1β treatment for 0–48 h. Data were considered significant if *p* = <0.05 (*) and *p* = <0.01 (**) following Dunnett's multiple comparison tests.

In a follow-up to this, the levels of CCL2 and IL-6 (both of which showed increased levels of translation and protein secretion in response to IL-1β exposure) were measured by ELISA in the media of either SW1353 cells or primary human articular chondrocytes (HACs) exposed to different doses of IL-1β for 24 h or for different times following stimulation with 10 ng/ml IL-1β (Fig. 3). Interestingly, the primary cells were more responsive to lower doses of IL-1β. This was particularly striking in the CCL2 data where the lowest concentration of IL-1β (0.1 ng/ml; Fig. 3*B*) was able to elicit a significant increase in CCL2 secretion, whereas SW1353 secretion only increased at 5 ng/ml and above (Fig. 3*A*). A similar result was found for the IL-6 data (Fig. 3, *E* and *F*), although variance of donor response for the primary chondrocytes precludes statistical confirmation of this with the current sample size. For both CCL2 and IL-6, the SW1353 cells (Fig. 3, *C* and *G*) and the primary chondrocytes (Fig. 3, *D* and *H*) exhibited similar longitudinal secretory responses to 10 ng/ml IL-1β, the experimental concentration used in Ribo-Seq and proteomic parts of the study.

### IL-1β induces altered translation of inflammatory-associated transcripts and translation via multiple open reading frames

**Figure 4.**
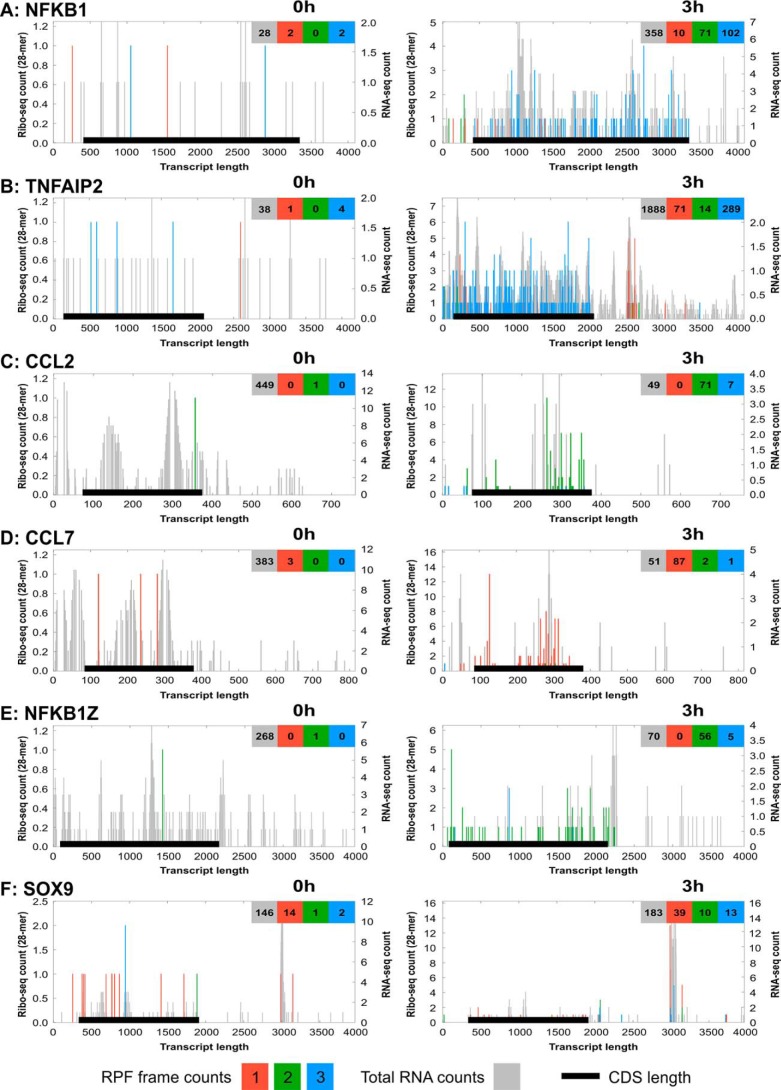
**Riboplots of differentially translated mRNA transcripts.** Using the ribo-Seq alignment files generated from aligning the sequencing data to the human transcriptome, riboplots were generated using the RiboPlot suite of RiboGalaxy for transcripts that were differentially translated between 0- and 3-h IL-1β exposure. Riboplots show the subcodon ribosome footprint and the ORF organization for both the 0- and 3-h time points for NFKB1 (GenBank^TM^ accession number NM_003998.3) (*A*), TNFAIP2 (NM_006291) (*B*), CCL2 (NM_002982.3) (*C*), CCL7 (NM_006273.3) (*D*), NFKB1Z (NM_031419.3) (*E*), and SOX9 (NM_000346.3) (*F*). Ribo-counts are shown for frame 1 (*red*), frame 2 (*green*), and frame 3 (*blue*). Total RNA reads in the background (*gray*) represent the total mRNA for all frames of the respective transcript. The *black lines* represent the CDS position within the transcript. The *x axis* represents transcript length (in nucleotides) 5′ to 3′. The *left y axis* represents the ribo-Seq counts for all three reading frames (RPFs). The *right y axis* represents the RNA-Seq counts for total RNA.

We examined Ribo-Seq and total RNA-Seq reads for differentially translated genes. Reads from each reading frame (measured against the *left-hand y axis*) were plotted on top of total RNA-Seq reads (plotted against the *right-hand y axis*) using the RiboGalaxy platform (Fig. 4). The *x axis* of these charts represents the entire length of the particular transcript with the coding domain sequence indicated. Some of the differentially translated transcripts examined, *e.g.* NFKB1 (Fig. 4*A*) and TNFAIP2 (Fig. 4*B*), exhibited an increase in both the total RNA counts and ribo-counts, suggesting an increase in both transcription and translation. Interestingly, some of the differentially translated transcripts exhibited a marked reduction in total RNA reads while simultaneously showing an increase in RPF reads, indicating reduced transcription rates and/or increased rates of turnover alongside an increased rate of translation. Examples of this were found for the chemokines CCL2 and CCL7 (Fig. 4, *C* and *D*) and the NF-κB signaling component NFKB1Z (Fig. 4*E*). We also observed clustered ribosome-protected reads outside of the normal transcript protein coding sequence (CDS), within discrete regions of the 3′-UTR, for a limited set of genes. Examples of these were reads observed in the 3′-UTR for TNFAIP2 (Fig. 4*B*) and for SOX9 (Fig. 4*F*) (although note that SOX9 was only in the top 200 differentially translated transcripts when all three reading frames were examined together and not when focusing on frame 1 from the triplet periodicity data). For SOX9, the increase in ribo-counts came mostly from regions within the SOX9 3′-UTR, suggesting that translation of SOX9 was not increased, but instead there was an increase in ribosome occupation or interaction at this region in response to IL-1β stimulation.

Despite the triplet periodicity and metagene analysis of 28-mer RPFs showing that the majority of transcripts were from protein coding regions within frame 1 (Fig. 1, *B* and *C*), individual riboplots for some transcripts that were identified as being differentially translated show that transcripts were being translated from more than one open reading frame (ORF). For example, the riboplot for CCL2 (Fig. 4*C*) shows that after IL-1β treatment there was an increase in CCL2 ribo-counts in frames 2 and 3; whereas for CCL7 (Fig. 4*D*), the majority of ribo-counts increased after IL-1β treatment were from frame 1 with just a couple from frames 2 and 3. Taken together, these results suggest that although the number of mRNA transcripts may increase or decrease in response to IL-1β treatment, rates of active mRNA translation do not necessarily reflect changes in transcription.

### IL-1β induces redox changes in SW1353 cells

**Figure 5.**
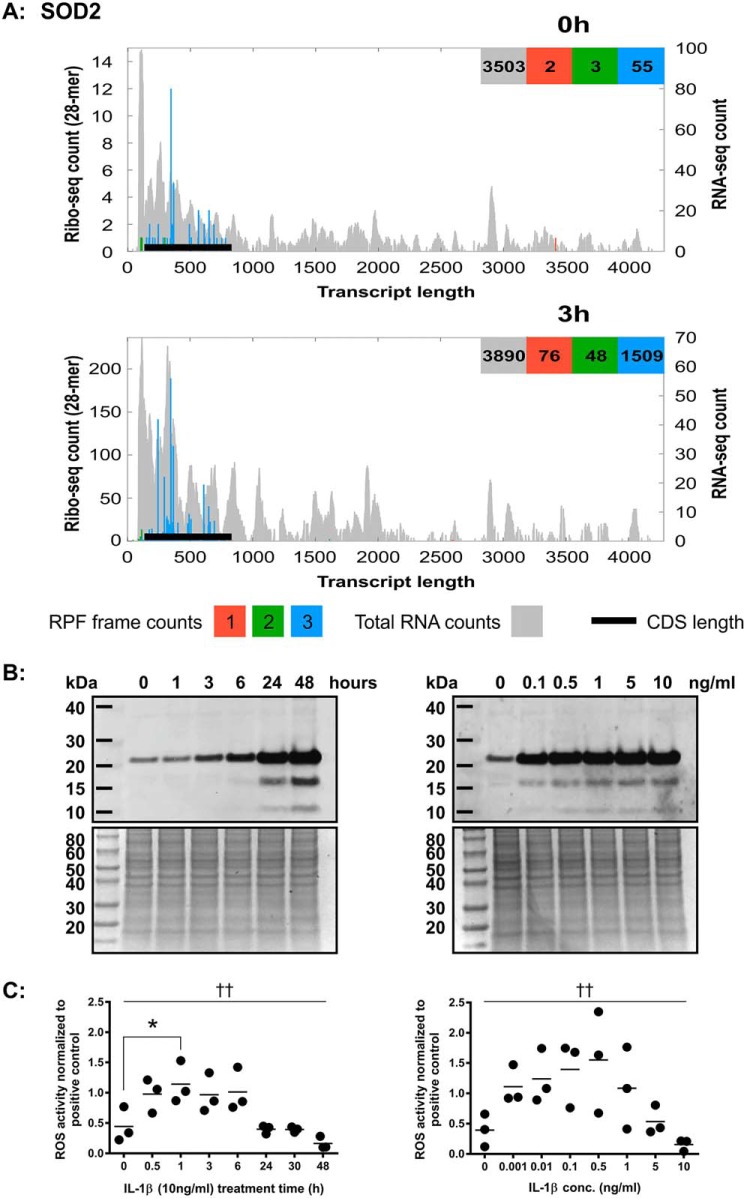
**IL-1β treatment causes an increase in SOD2 mRNA and protein expression in SW1353 cells.** *A*, riboplot for SOD2 (GenBank accession number NM_000636.3). Ribo-counts are shown for frame 1 (*red*), frame 2 (*green*), and frame 3 (*blue*). Total RNA reads in the background (*gray*) represent the total mRNA for all frames of the respective transcript. The *black lines* represent the CDS position within the transcript. The *x axis* represents transcript length (in nucleotides). The *left y axis* represents the ribo-Seq counts for all three reading frames. The *right y axis* represents the RNA-Seq counts for total RNA. *B*, Western blot for SOD2 (22 kDa) in SW1353 intracellular protein lysates treated with 10 ng/ml IL-1β for 0–48 h (*left*, *top*) and corresponding total protein stain (*left*, *bottom*) plus Western blot for SOD2 in SW1353 intracellular protein lysates treated with 0–10 ng/ml IL-1β for 24 h (*right*, *top*) and corresponding total protein stain (*right*, *bottom*). *C*, CM-H_2_DCFDA assay for SW1353 cells treated with 10 ng/ml IL-1β for 0–8 h (*left*) and treated with 0–10 ng/ml IL-1β for 24 h (*right*). Data variance was determined by ANOVA (*p* < 0.05 (†) or *p* < 0.01 (††)). *, significant difference *versus* 0-h treatment time following Dunnett's multiple comparison testing (*p* < 0.05).

Results from the SW1353 cellular proteomic data showed that SOD2 was one of the highest ranked, differentially expressed proteins following exposure to IL-1β (Table S1). When ribosome profiling data from all three frames were analyzed, SOD2 was identified as one of the most highly differentially translated transcripts The riboplot for SOD2 (Fig. 5*A*) showed that SOD2 reads were predominantly in frame 3 and, of all the transcripts examined, show the largest increase in ribo-counts following IL-1β treatment (from 55 to 1509). Total RNA read coverage for SOD2 remained largely unchanged, however, strongly suggesting that IL-1β significantly increases SOD2 levels at the translational level. Western blotting of cellular lysates from IL-1β (10 ng/ml)–treated SW1353 showed that this increase in translation also resulted in an increase in protein expression in a time-dependent manner (Fig. 5*B*, *left panel*). After 24 h of IL-1β stimulation at a range of IL-1β concentrations, SOD2 levels were also increased (Fig. 5*B*, *right panel*). However, it is currently unknown at what timeframe this SOD2 expression took place.

To further investigate the possibility that IL-1β treatment causes redox changes, reactive oxygen species (ROS) detection assays were carried out. Results show that with 10 ng/ml IL-1β treatment there is a significant level of variance of ROS activity over time (Fig. 5*C*, *left panel*). IL-1β treatment appears to quickly induce levels of ROS, which then are reduced with time, in correlation to SOD2 protein expression via Western blotting (Figs 5, *B* and *C*, *left panels*). Concentrations of IL-1β as low as 0.1 ng/ml resulted in an increase in ROS activity (Fig. 5*C*, *right panel*) but still induced SOD2 at similar levels to the 10 ng/ml dose after 24 h. These data along with the increase in NFKB1 mRNA expression and translation (Fig. 4*A*) suggest that IL-1β treatment activates NF-κB and a cellular stress response within the SW1353 cells that is controlled through translational mechanisms.

## Discussion

To study how IL-1β alters translation using ribosome profiling, a suitable model system was required that maintained biologically relevant aspects of cell function with the practical requirements of obtaining relatively large quantities of total RNA. The number of cells required for ribosome profiling made the use of freshly isolated primary chondrocytes unfeasible, whereas expansion of these cells results in a progressive loss of phenotype that quickly leads to them sharing the majority of their transcriptomic signature with cultured cells from connective tissues such as tendon (39). We were therefore attracted to the use of a chondrocytic cell line that exhibited similar responsiveness to IL-1β as primary chondrocytes. Gebauer *et al.* (13) found that the SW1353 chondrosarcoma cell line displayed considerably reduced expression of extracellular matrix components but were still a valuable system for investigating catabolic gene regulation by IL-1β. In line with this, our data have shown that there is an increase in IL-1β, IL-6, LIF, NFKB1, and TNFAIP6 mRNA expression following IL-1β stimulation (Table S5) that replicates that observed in IL-1β–treated HACs by Gebauer *et al.* (13). There are always limitations to accept with such cell lines of course, and there is evidence that SW1353 and another chondrocyte cell line, T/C-28a2, do not regulate inducible nitric-oxide synthase expression in the same way as HACs following stimulation with proinflammatory cytokines (40). However, in this instance, we decided to use the SW1353 cell line as an *in vitro* replacement for HACs.

We believe that this is the first time that ribosome profiling has been used to study a chondrocytic cell type. Ribosome-protected fragments were detected from 26 to 30 nucleotides in length with the majority being 28-mers (Fig. 1*B*). Metagene analysis with our ribosome profiling data showed that most RPFs were found relatively evenly across protein coding regions as expected. However, there were instances where RPFs were found within the 3′-UTR such as in TNFAIP2 (Fig. 4*B*) and SOX9 (Fig. 4*F*). Miettinen and Björklund (41) suggest that this association of ribosomes with the 3′-UTR could be due to migration of the ribosome through the stop codon or the 3′-UTR folding around on itself and associating with ribosomes on the CDS. Therefore, the potential for interactions with ribosomes and the 3′-UTR of these mRNAs suggests a role in translational regulation. Given the well-established role of these two proteins in cartilage biology, this may represent an interesting new regulatory mechanism controlling cartilage function.

Although it appears from our complementary proteomic and ribosome profiling data that IL-1β treatment results in an up-regulation of ribosome-associated transcripts and transcripts for inflammatory mediators, the presence of ribosome-associated proteins in the SW1353 secretome at first seemed unusual. However, the source of ribosome-associated proteins in the media samples could be due to the presence of exosomes, which are constantly secreted by all cells in culture. Exosomes are small vesicles (30–150 nm) containing up to thousands of proteins and small RNAs, and they have a variety of functions such as facilitation of the immune response, antigen presentation, programmed cell death, and inflammation (42). Exosomes are also a novel mechanism for cell-to-cell communication (43). Another possibility for the source of ribosome-associated proteins in the cell culture media is due to cell lysis at the point of media harvesting, and the presence of actin and other actin-related proteins (Table S2) supports this theory. There was also a large proportion of ribosome-associated transcripts identified through ribosome profiling differential translation analysis. It is not clear whether this change is a specific response to IL-1β or a general response induced by a potential alteration in the translational burden on the cells during their response.

Differential translation analysis in this study showed that as well as ribosome-associated and inflammatory mediator–associated transcripts being up-regulated in response to IL-1β treatment, the expression of 14 pseudogenes, nine of which were pseudogenes for ribosomal proteins, was also detected. Ribosomal protein pseudogenes represent the largest class of pseudogenes within the human genome (44), and ribosomal pseudogenes have been detected in many human tissues, but their function remains unclear (45). Differential translation analysis also found that IL-1β treatment decreased the translation of only two transcripts: PIM1 and ZC3H12A. PIM1 is a proto-oncogene with serine/threonine kinase activity involved in cell survival, proliferation, and apoptosis (46). As exogenous IL-1β treatment has been shown to both stimulate and inhibit apoptosis in different cell systems (47), the decrease in PIM1 expression seen here could suggest that IL-1β treatment could be activating apoptosis in this system, although no other apoptosis-associated transcripts were detected in the differential translation analysis. ZC3H12A is a transcriptional activator with endoribonuclease activity that is involved in a variety of biological functions, including the cellular inflammatory response. IL-1β has been shown to up-regulate ZC3H12A expression via the NF-κB and extracellular signal-regulated kinase pathways (48), which is contrary to what was found in this study.

The riboplots in Fig. 4 show that some transcripts such as NFKB1 and TNFAIP2 experience an increase in transcript level and translation rate following IL-1β exposure, whereas other transcripts such as CCL2, CCL7, and NFKB1Z experience a simultaneous reduction in transcript level with an increase in translation. This observation of genes under both transcriptional and translational control has also been observed by others (49, 50) and confirms the complexity of gene regulation. Elevation of CCL2 and CCL7 in particular represents a primed, early response to IL-1β stimulation that is likely to be characterized by rapid secretion that will be quickly switched off. The role of the rapid, translationally mediated secretion of chemokines by chondrocytes may represent an important, physiological means of cell-to-cell signaling in healthy tissue and merits further study.

OA was once considered to be a noninflammatory disease caused by excessive wear and tear on the articular cartilage of the affected joint. More recently, however, it has been recognized that inflammation plays a significant role in OA pathogenesis (7, 8, 51). In both diseased tissue and in this *in vitro* model, the onset of inflammation by inflammatory cytokines such as IL-1β up-regulates the expression of transcripts that code for enzymes such as MMP13, which causes further cartilage degradation (52). ROS and oxidative stress have also been shown to play a significant role in OA progression (53, 54), and through the activation of the redox-sensitive NF-κB transcription factor, chondrocytes increase SOD2 production to prevent ROS-mediated damage (55–57). Across the three areas studied here, cellular proteome, secretome, and translatome, activation of NF-κB in response to IL-1β was consistent. The increase in SOD2 translation and protein expression in this SW1353 model suggests that IL-1β induces changes in the mitochondrial redox balance through translational regulation. ROS levels rise rapidly upon IL-1β stimulation and drop as production of SOD2 rises. However, low levels of IL-1β (*e.g.* 0.1 ng/ml) can lead to increased ROS generation at 24 h despite SOD2 levels increasing, suggesting that there is a greater complexity to the mechanism of cytokine-responsive redox control in these cells.

The mechanisms through which chondrocyte translation is regulated have not been thoroughly studied. However, two pioneering recent studies have shown that osteoarthritic and cytokine-mediated translational changes in chondrocytes are mediated by the activity of the cap-dependent translation repressor 4E-binding protein 1 (33), possibly acting downstream of mTOR complex 1 signaling (59). In addition, small RNAs that are known to regulate translation such as microRNAs and small nucleolar RNAs are known to be differentially expressed in normal and osteoarthritic cartilage (29, 61). The influences of these mechanisms on the translation of many of the transcripts identified in this study are clearly now warranted.

In summary, this study shows for the first time the use of ribosome profiling in a chondrocytic cell, observing the effects of IL-1β on translational regulation. We have shown that through differential translation IL-1β can induce the secretion of inflammatory proteins and promote changes in redox regulators in these cells. It is possible that chondrocytes are primed to rapidly respond to inflammatory cytokine stimulation, producing a cascade of further inflammatory mediators. Whether there is a role for this rapid responsiveness in the maintenance of tissue homeostasis in healthy tissue remains, for now, a key unanswered question.

## Experimental procedures

### Cell culture

The SW1353 chondrosarcoma cell line (ATCC HTB-94) was cultured in Dulbecco's modified Eagle's medium (DMEM; Thermo Fisher Scientific, 31885023) containing 10% fetal bovine serum (Sigma, F7524), 100 units/ml penicillin-streptomycin (Gibco, 15140122), and 5 μg/ml amphotericin B (Gibco, 15290018) at 37 °C in a 5% CO_2_ environment. Osteoarthritic human articular cartilage was obtained with approval from the Cheshire Research Ethics Committee following total knee arthroplasty. Primary articular chondrocytes were isolated from cartilage tissue using the medium described above supplemented with 0.08% type II collagenase (Worthington Biochemical Corp.) overnight at 37 °C. Cells were cultured as described for SW1353.

### Proteomic MS and label-free quantification

To determine how protein secretion and cellular proteome changes were affected by cytokine stimulation, SW1353 cells were cultured for 24 h in the presence or absence of 10 ng/ml IL-1β in serum-free, phenol red-free DMEM (*n* = 3 for each condition). Medium was collected from the cells and then subjected to in-solution trypsin digestion and LC-MS/MS (see below). Protein extracts from the SW135 cell layer were prepared in 50 mm ammonium bicarbonate containing 25 mm *N*-ethylmaleimide (*d*_0_-NEM), pH 8. Protein lysates were prepared by centrifugation at 15,000 × *g* for 10 min at 4 °C. Excess *d*_0_-NEM was removed using Zeba desalting columns (Thermo Fisher Scientific), and protein concentrations were determined using a Bradford assay (Bio-Rad) with BSA as a standard. 100 μg of protein extract was diluted to 160 μl with 25 mm ammonium bicarbonate and denatured by the addition of 10 μl of 1% RapiGest (Waters, Manchester, UK) in 25 mm ammonium bicarbonate and incubated at 80 °C for 10 min with shaking. 10 μl of a 100 mm solution of tris(2-carboxyethyl)phosphine hydrochloride was added to reduce reversibly oxidized Cys residues followed by incubation at 60 °C for 10 min. Newly reduced Cys residues were then alkylated by addition of *d*_5_-NEM and incubated at room temperature for 30 min. An aliquot of the samples was used at this point to check the procedure by SDS-PAGE. Proteolytic digestion was performed by addition of trypsin followed by overnight incubation at 37 °C. Digestion was terminated, and RapiGest was removed by acidification (3 μl of TFA and incubation at 37 °C for 45 min) and centrifugation (15,000 × *g* for 15 min).

Mass spectrometry was performed using an Ultimate 3000 RSLC^TM^ Nano system (Thermo Fisher Scientific) coupled to a QExactive^TM^ mass spectrometer (Thermo Fisher Scientific). 250 ng per sample was loaded onto the trapping column (Thermo Scientific, PepMap100, C_18_, 75 μm × 20 mm), using partial loop injection, for 7 min at a flow rate of 4 μl/min with 0.1% (v/v) TFA. The sample was resolved on the analytical column (Easy-Spray, C_18_, 75 μm × 500 mm, 2-μm column) using a gradient of 97% A (0.1% formic acid), 3% B (99.9% acetonitrile, 0.1% formic acid) to 60% A, 40% B over 120 min at a flow rate of 300 nl/min. The program used for data acquisition consisted of a 70,000-resolution full-scan MS scan (automatic gain control set to 10^6^ ions with a maximum fill time of 250 ms). The 10 most abundant peaks were selected for MS/MS using a 17,000-resolution scan (automatic gain control set to 5 × 10^4^ ions with a maximum fill time of 250 ms) with an ion selection window of 3 *m*/*z* and a normalized collision energy of 30. To avoid repeated selection of peptides for MS/MS, the program used a 30-s dynamic exclusion window.

Label-free relative quantification software PEAKS^TM^ 7 (Bioinformatics Solutions Inc., Waterloo, Canada) was used to analyze RAW data files against the same mouse protein database for identifications with Mascot. Proteins were considered significantly changed between 0- and 24-h IL-1β–treated samples using a −10log *p* score >20 (equivalent to a *p* value <0.01), a -fold change >2, and at least three unique peptides. The full list of identified proteins, including statistical analysis, is included in Table S1.

Protein concentrations of medium samples from SW1353 cultured for 24 h in the presence or absence of 10 ng/ml IL-1β in serum-free, phenol red-free DMEM (*n* = 3 for each condition) were estimated by Bradford assay using Coomassie Plus^TM^ protein assay reagent (Thermo Fisher Scientific, 23236) read at 600 nm. Prior to trypsin digestion, the protein concentration of each sample was calculated using the Pierce^TM^ 660 nm protein assay (Thermo Fisher Scientific, 22662). In-solution tryptic digestion was carried out on 10 μl of StrataClean resins (Agilent Genomics, 400714) on 100 μg of protein for each sample as described previously (62). LC-MS/MS was performed using an Ultimate 3000 RSLC Nano system coupled to a QExactive mass spectrometer. Tryptic peptides (250 ng) from randomized samples were loaded onto the column on a 2-h gradient with an intersample 30-min blank (63).

### Western blotting

For Western blotting, homogenized protein lysates were diluted using Laemmli buffer, and 20 μg of protein was separated by 12% SDS-PAGE. Proteins were transferred using a semidry blotter. Membranes were blocked in Odyssey blocking buffer in Tris-buffered saline (LI-COR Biosciences, 927-50000) and then incubated with primary antibody for SOD2 (Cell Signaling Technology, 13194) at a dilution of 1:750 in blocking buffer. IRDye 800CW goat anti-rabbit secondary antibody (LI-COR Biosciences, 925-32210) was diluted 1:10,000 in blocking buffer. Antibody signal was detected using the Odyssey CLx imaging system (LI-COR Biosciences), and images were acquired and analyzed using Image Studio acquisition software (LI-COR Biosciences). Total protein staining was carried out using InstantBlue Protein Gel Stain (Expedeon, ISB1L) and then imaged using a ChemiDoc XBS+ molecular imager (Bio-Rad) and Image Lab software (Bio-Rad).

### CCL2 and IL-6 ELISAs

SW1353 and HACs were treated with 0, 0.1, 0.5, 1, 5, and 10 ng/ml IL-1β for 24 h or with 10 ng/ml IL-1β for 0, 1, 3, 6, 24, and 48 h, and medium was harvested for CCL2 (R&D Systems, DY279) and IL-6 (R&D Systems, DY206) ELISAs. For IL-1β concentration-dependent treatment, HAC donors M70, M76, and M86 were used. For time-dependent IL-1β treatment, HAC donors M69, F80, and F86 were used. Optical density was measured using a SPECTROstar Nano spectrophotometer (BMG Labtech), and MARS data analysis software (BMG Labtech). Cytokine secretion levels were normalized to either 0 ng/ml IL-1β or 0-h treatment. Charts were plotted and statistical analysis (ANOVA and Dunnett's multiple comparison test) was performed using Prism (GraphPad Software). Data were considered statistically significant if the *p* value was <0.05.

### ROS detection assay

For ROS detection, SW1353 cells were treated with IL-1β (ranging from 0 to 10 ng/ml) for 24 h or with 10 ng/ml IL-1β for 0, 0.5, 1, 3, 6, 24, 30, and 48 h in 96-well plates. H_2_O_2_ treatment (200 μm for 2 h) served as a positive control for ROS production. ROS detection was carried out using CM-H_2_DCFDA (Thermo Fisher Scientific, C6827). Fluorescence was measured using a SPECTROstar Nano spectrophotometer and MARS data analysis software. Charts were plotted, and statistical analysis (Dunnett's multiple comparison test) was performed using Prism. Data were considered statistically significant if the *p* value was <0.05.

### Purifying ribosome-protected RNA

Ribosome profiling was carried out similarly to the method described by Ingolia *et al.* (58), modified with the use of an ARTseq (Mammalian) Ribosome Profiling kit (Epicenter, RPHMR12126). Serum was withdrawn when the cells had reached ∼80% confluence, and then 24 h later the cells were treated with 10 ng/ml IL-1β (Sigma, I9401) for 3 and 24 h (3 × 175-cm^2^ flasks per time point, *n* = 3).

To harvest cells for ribosome profiling, cell culture medium was removed, and cells were washed with ice-cold PBS containing 0.1 mg/ml cycloheximide (Millipore UK Ltd., 239763). Cells were scraped into 800 μl of mammalian lysis buffer (20% (v/v) 5× Mammalian Polysome Buffer (supplied in the ARTseq Ribosome Profiling kit), 1% (v/v) Triton X-100 (supplied), 10 mm DTT (supplied), 10 units/μl DNase I (supplied), 0.1 mg/ml cycloheximide) and then passed through a 22–25-gauge needle to lyse the cells completely. Cell lysate was then incubated on ice for 10 min and then centrifuged for 10 min at 20,000 × *g* at 4 °C. The supernatant was transferred to a prechilled tube and kept on ice. A 1:10 dilution of the clarified supernatant was prepared using nuclease-free water, and the *A*_260_ reading of the lysate was determined using a NanoDrop spectrophotometer. For each sample, the supernatant was split into 100-μl aliquots, and to one aliquot, 10 μl of 10% SDS was added and served as the “total RNA” sample. To the remaining 100-μl aliquots, 5 units of ARTseq nuclease was added for each *A*_260_ of lysate and incubated at room temperature for 45 min with gentle mixing. Nuclease digest reactions were stopped by the addition of 300 units of SUPERase·In inhibitor (Thermo Fisher Scientific, AM2696). The RPFs were purified according to the ARTseq (Mammalian) Ribosome Profiling kit protocol. Briefly, the 100-μl aliquots that were nuclease-digested earlier were added to Micro-Spin S-400 columns (GE Healthcare, 27-5140-01), which had been equilibrated by gravity flow with 3 ml of 1× Mammalian Polysome Buffer and centrifuged for 2 min at 600 × *g*. The flow-through was collected, and 10 μl of 10% SDS was added; this then served as the RPF RNA sample.

### RNA purification and ribosome depletion

Total and RPF RNA samples were purified using the TRIzol/chloroform method. rRNA was depleted from the total and RPF RNA samples using a Ribo-Zero rRNA Removal kit (Epicenter, RHZ110424). Briefly, 1–5 μg of RNA was incubated at 68 °C for 10 min with Ribo-Zero rRNA Removal Solution (supplied) and then at room temperature for 15 min before mixing with pre-prepared microspheres (supplied). Hybridized RNA–microspheres were incubated at room temperature with frequent mixing for 10 min to allow rRNA to bind to the microspheres, which were then removed by centrifuging the sample at 12,000 × *g* in a Microsphere Removal Unit (supplied). The filtrate containing the rRNA-depleted RNA was then purified using the TRIzol/chloroform protocol described above. rRNA-depleted total RNA samples were then heat-fragmented (see below), and RPF RNA samples were then PAGE-purified.

### Fragment and end repair and 3′ adaptor ligation

rRNA-depleted total RNA samples were heat-fragmented at 94 °C for 20 min and then held at 4 °C. Both heat-fragmented total RNA samples and PAGE-purified RPF RNA samples were then end-repaired using ARTseq polynucleotide kinase enzyme, incubating for 1 h at 37 °C. RNA samples were then purified using the TRIzol/chloroform protocol and eluted into 10 μl of nuclease-free water. RNA samples were incubated with the supplied ARTseq 3′ adaptor (supplied) for 2 min at 65 °C and then held at 4 °C. These denatured RNA samples were then incubated with the supplied ARTseq ligase (supplied), ligation buffer (supplied), and 100 mm DTT at 23 °C for 2 h. The ARTseq adaptor removal enzyme (supplied) was then added to this ligation mixture and incubated at 30 °C for 30 min. RNA samples were then reverse transcribed.

### cDNA synthesis and circularization

The 3′ adaptor–ligated RNA samples were reverse transcribed using EpiScript reverse transcriptase (supplied) at 50 °C for 30 min. Reverse transcription samples were then incubated with the ARTseq exonuclease (supplied) at 37 °C for 30 min and then 80 °C for 15 min before adding the ARTseq RNase mixture (supplied) and incubating the samples at 55 °C for 5 min. Samples were then held at 4 °C before PAGE purifying the cDNAs on 10% polyacrylamide, 7–8 m urea, Tris borate-EDTA gels using bromphenol blue at 180 V with SYBR Gold (Thermo Fisher Scientific, S11494) staining. cDNA was circularized using CircLigase (supplied) by incubating for 2 h at 60 °C with the ARTseq CircLigase Reaction Mix (supplied), ATP, and MnCl_2_ (both supplied) before holding at 4 °C. Circularized cDNA was then PCR-amplified as described below.

### PCR amplification

Circularized cDNA (5 μl) was mixed with 2 μl of the ARTseq forward PCR primer (supplied), 2 μl of a ScriptMiner Index PCR primer of choice (supplied index primers 1–12), and 25 μl of 2× Phusion High-Fidelity PCR Master Mix (New England Biolabs, M0531) in a total volume of 50 μl. Samples were then run in the following PCR program: 98 °C for 30 s and then 15 cycles of 94 °C for 15 s, 55 °C for 5 s, and 65 °C for 10 s. PCR products were purified using Agencourt AMPure XP beads (Beckman Coulter, A63880) according to the manufacturer's instructions and eluted into 25 μl of nuclease-free water. To check for successful PCR amplification, 2.5 μl of the PCR-amplified samples was mixed with 6× native gel loading dye and run on a Novex 8% native Tris borate-EDTA gel (Thermo Fisher Scientific, EC6215BOX) at 200 V until the dye front reached the bottom of the gel with ΦX174 RF DNA/HaeIII fragments as markers (Thermo Fisher Scientific, 15611-015). The gel was then stained with SYBR Gold and visualized using a dark-field transilluminator. Samples that contained successfully amplified libraries (140–160 bp) were sent for quality control and sequencing.

### Quality control and sequencing

Successfully amplified PCR libraries were sent to the University of Liverpool's Centre for Genomic Research for sequencing. Samples were quantified using a Qubit dsDNA High Sensitivity kit (Life Technologies, Q32854) and a Bioanalyzer DNA High Sensitivity kit (Agilent, 5067-4626) using 1 μl per sample of a 2 ng/μl dilution of each library. Libraries were then pooled at equimolar quantities, and the pooled libraries were size-selected within 120–160 bp on a SAGE PippinPrep instrument using a 1.5% agarose gel cassette. The size-selected pooled library was assessed by Bioanalyzer and subsequently by quantitative PCR using an Illumina Library Quantification kit (KAPA Biosystems, KK4854) on a Roche Applied Science Light Cycler LC480II according to the manufacturer's instructions. The template DNA was denatured according to the protocol described in the Illumina cBot user guide and loaded a at 12 pm concentration. Sequencing was performed using an Illumina HiSeq2500 with 125-bp reads at the Centre for Genome Research at the University of Liverpool. A summary of the quality control data can be found in Table S3.

### RNA-Seq data analysis

The total number of reads from each sample varied from 7.5 × 10^6^ to 5.9 × 10^7^. FASTQ files containing reads from ribosome-protected and total RNA samples were uploaded to the RiboGalaxy server where sequences were first trimmed to remove adaptor sequences. Following this, rRNA reads were removed from the data, and then riboSeqR was used via the RiboGalaxy interface to align remaining reads to the human genome (GRCh38) before performing differential translation analysis (60). A detailed description of the workflow and RiboGalaxy parameters can be found in Fig. S1 and Table S4, respectively.

### Functional gene characterization and protein networks

Ribosome profiling and proteomic data were submitted to the Database for Annotation, Visualization and Integrated Discovery (DAVID) online analysis program (version 6.8, https://david.ncifcrf.gov) (64, 65). Functional and physical interactions between proteins were discovered using the online STRING Protein-Protein Interaction Network (https://string-db.org/)^3^ (66).

### Data deposition

The RNA-Seq data representing both total and ribosome-protected RNA have been deposited in ArrayExpress (accession number E-MTAB-7466). Both cellular proteomic and secretome data have been deposited in PRIDE (accession number PXD012985).

## Author contributions

B. T. M. and S. R. T. conceptualization; B. T. M., M. J. P., B. M., and S. R. T. data curation; B. T. M., M. J. P., B. M., and S. R. T. formal analysis; B. T. M. investigation; B. T. M., M. J. P., and B. M. methodology; B. T. M. and S. R. T. writing-original draft; B. T. M. and S. R. T. writing-review and editing; M. J. P., B. M., and S. R. T. resources; B. M. software; S. R. T. supervision; S. R. T. validation; S. R. T. visualization.

## Supplementary Material

Supporting Information

## Footnotes

3 Please note that the JBC is not responsible for the long-term archiving and maintenance of this site or any other third party hosted site.

## Footnotes

2 The abbreviations used are:

OA: osteoarthritis
CCL: chemokine (CC motif) ligand
CDS: coding sequence
HAC: human articular chondrocyte
IL: interleukin
MMP: matrix metalloproteinase
NEM: *N*-ethylmaleimide
RPF: ribosome-protected fragment
ROS: reactive oxygen species
SOD: superoxide dismutase
STRING: Search Tool for the Retrieval of Interacting Genes/Proteins
TNFα: tumor necrosis factor α
TNFAIP: TNFα-induced protein
DMEM: Dulbecco's modified Eagle's medium
ANOVA: analysis of variance
CM-H_2_DCFDA: 5-(and-6)-chloromethyl-2′,7′-dichlorodihydrofluorescein diacetate
PKM: pyruvate kinase
LIF: leukemia inhibitory factor.
