# Supplementary material for: Translational regulation contributes to the secretory response of chondrocytic cells following exposure to interleukin-1β
Source: J Biol Chem. 2019 Jul 12;294(35):13027–39. doi: 10.1074/jbc.RA118.006865 (PMC6721953; doi:10.1074/jbc.RA118.006865)
Supplement: Supporting Information [file supp_294_35_13027__index.html]

Translational regulation contributes to the secretory response of chondrocytic cells following exposure to Interleukin-1β — IL-1β induces translational regulation in chondrocytic cells — Translational regulation contributes to the secretory response of chondrocytic cells following exposure to interleukin-1β — IL-1β induces translational regulation in chondrocytic cells — Supporting Information 

# Translational regulation contributes to the secretory response of chondrocytic cells following exposure to interleukin-1β

## Supporting Information

- Supporting Information (to be published online) - Supporting information figures S1-S4
- Supporting Information tables S1 - S5 - Supporting information tables for proteomic data results, ribosome profiling differential translation analysis, and full set of RiboGalaxy parameters.
