# Supplementary material for: Translational regulation contributes to the secretory response of chondrocytic cells following exposure to interleukin-1β
Source: J Biol Chem. 2019 Jul 12;294(35):13027–39. doi: 10.1074/jbc.RA118.006865 (PMC6721953; doi:10.1074/jbc.RA118.006865)
Supplement: Supporting Information [file supp_RA118.006865_142182_1_supp_345305_psvhz8.pdf]

Translational regulation contributes to the secretory response of chondrocytic cells following exposure to Interleukin-1β

Benjamin T McDermott[1], Mandy J Peffers[1], Brian McDonagh[2] and Simon R Tew[1]

Supporting Information

Figure S1: Extended ribosome profiling workflow detailing steps that were performed on the RiboGalaxy platform.

Figure S2: SW1353 STRING analysis of ribosome profiling transcripts.

Figure S3: SW1353 STRING analysis of secretome data.

Figure S4: SW1353 STRING analysis of cellular proteomic data.

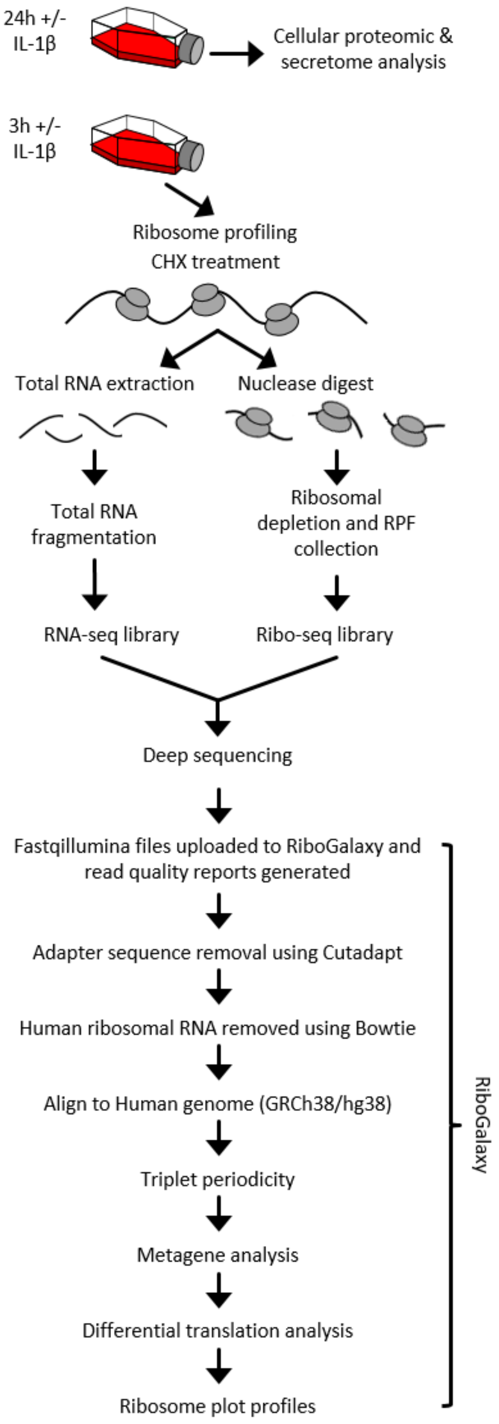

Fig. S1

Supporting Information Figure S1. Extended ribosome profiling workflow detailing steps that were performed on the RiboGalaxy platform. A full list of detailed parameters used in RiboGalaxy are described in Supporting Information Table S4.

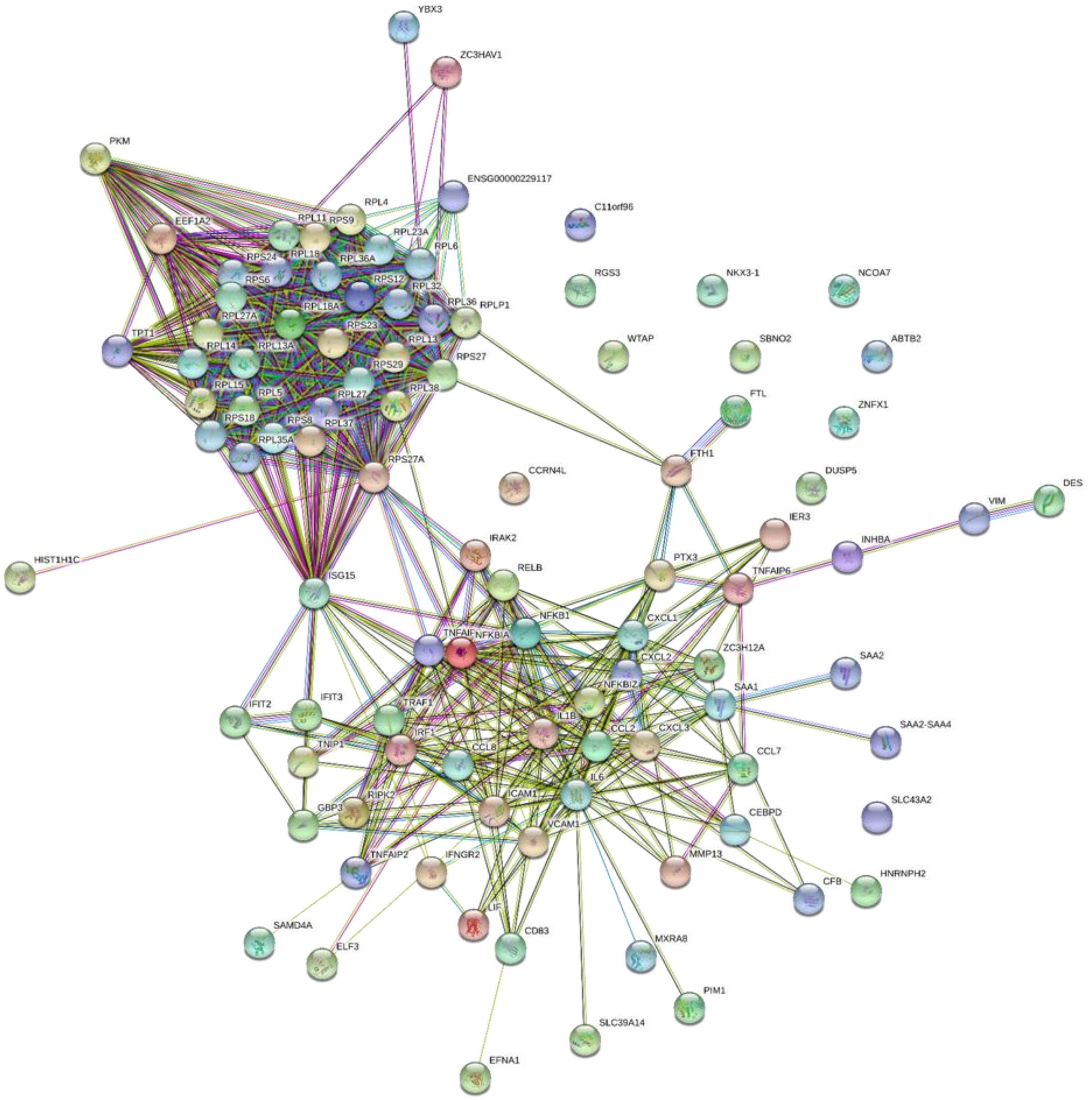

Fig. S2

Supporting Information Figure S2. SW1353 STRING analysis of ribosome profiling transcripts. A larger version of the STRING network shown in Figure 2A, with the addition of transcript labels.

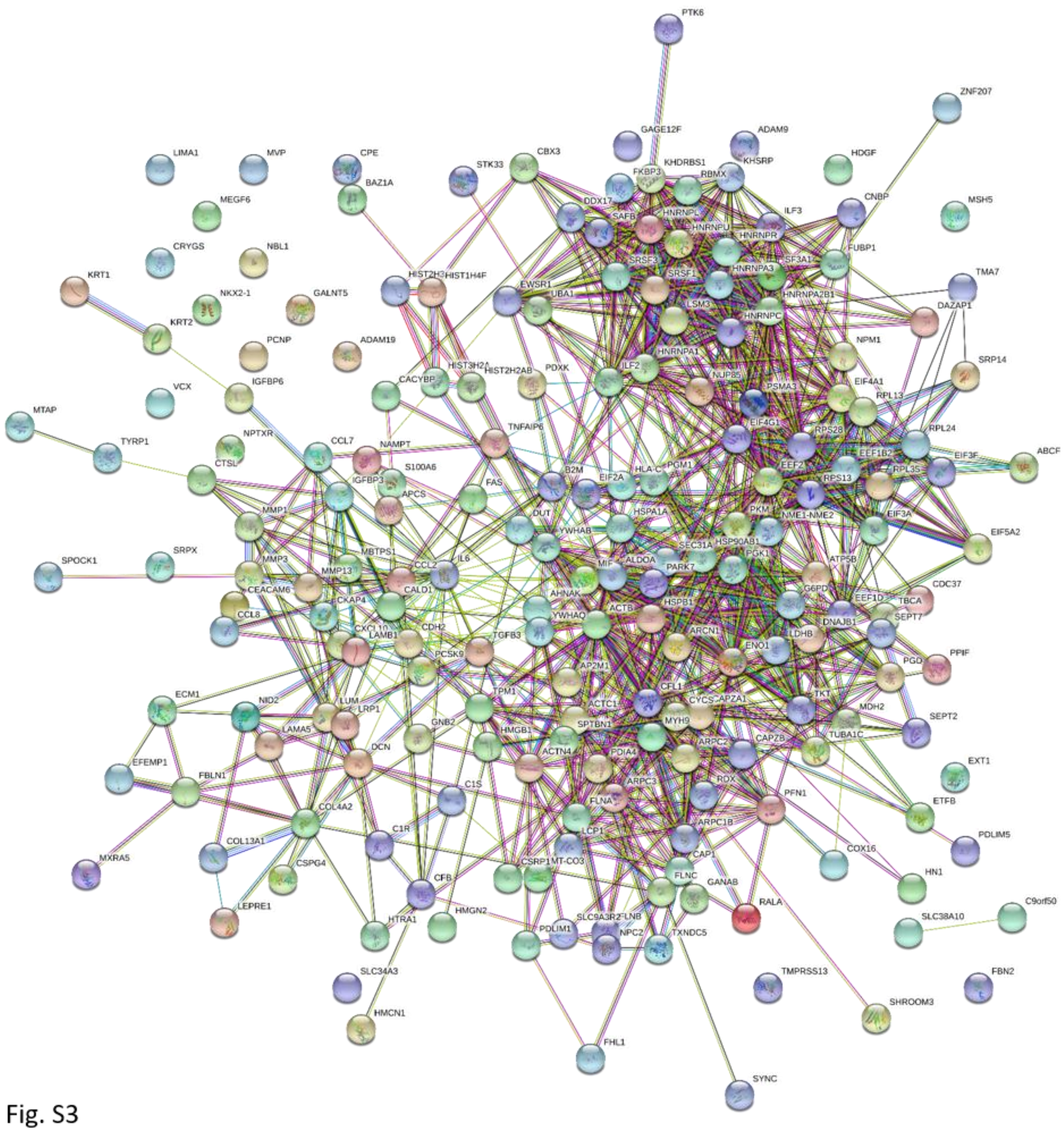

Fig. S3

Supporting Information Figure S3. SW1353 STRING analysis of secretome data. A larger version of the STRING network shown in Figure 2B, with the addition of transcript labels.

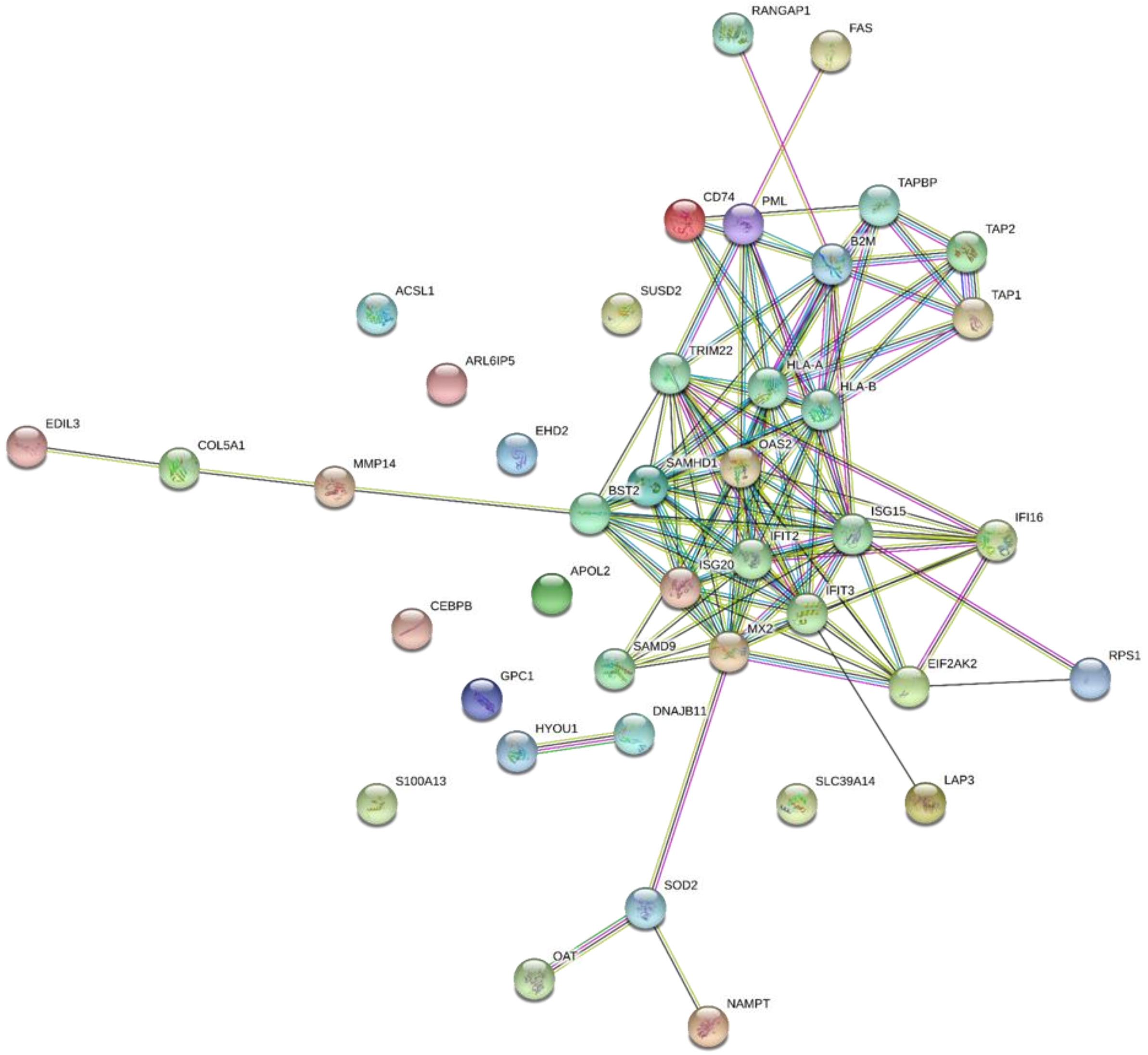

Fig. S4

Supporting Information Figure S4. SW1353 STRING analysis of cellular proteomic data. A larger version of the STRING network shown in Figure 2C, with the addition of transcript labels.
